# Screening and functional prediction of differentially expressed circRNAs in proliferative human aortic smooth muscle cells

**DOI:** 10.1111/jcmm.15150

**Published:** 2020-03-10

**Authors:** Wei Chen, Jiajie Lin, Bin Li, Shanhu Cao, Huanhuan Li, Jianzhi Zhao, Kun Liu, Yiming Li, Yang Li, Shaoguang Sun

**Affiliations:** ^1^ Department of Biochemistry and Molecular Biology Key Laboratory of Medical Biotechnology of Hebei Province Institute of Medicine and Health Hebei Medical University Shijiazhuang China; ^2^ Stem Cell Translational Research Center Tongji Hospital Tongji University School of Medicine Shanghai China

**Keywords:** circular RNAs, coding potential, HASMCs, microRNA sponge, proliferation

## Abstract

Vascular smooth muscle cell (VSMC) proliferation is the pathological base of vascular remodelling diseases. Circular RNAs (circRNAs) are important regulators involved in various biological processes. However, the function of circRNAs in VSMC proliferation regulation remains largely unknown. This study was conducted to identify the key differentially expressed circRNAs (DEcircRNAs) and predict their functions in human aortic smooth muscle cell (HASMC) proliferation. To achieve this, DEcircRNAs between proliferative and quiescent HASMCs were detected using a microarray, followed by quantitative real‐time RT‐PCR validation. A DEcircRNA‐miRNA‐DEmRNA network was constructed, and functional annotation was performed using Gene Ontology (GO) and KEGG pathway analysis. The function of hsa_circ_0002579 in HASMC proliferation was analysed by Western blot. The functional annotation of the DEcircRNA‐miRNA‐DEmRNA network indicated that the four DEcircRNAs might play roles in the TGF‐β receptor signalling pathway, Ras signalling pathway, AMPK signalling pathway and Wnt signalling pathway. Twenty‐seven DEcircRNAs with coding potential were screened. Hsa_circ_0002579 might be a pro‐proliferation factor of HASMC. Overall, our study identified the key DEcircRNAs between proliferative and quiescent HASMCs, which might provide new important clues for exploring the functions of circRNAs in vascular remodelling diseases.

## 1 INTRODUCTION

Cardiovascular diseases (CVD), including vascular remodelling diseases, are the leading cause of mortality among humans throughout the world. Abnormal proliferation of vascular smooth muscle cells (VSMCs) is a common feature of many vascular remodelling diseases, including atherosclerosis,^1^ hypertension^2^ and vascular aneurysms.^3^ The regulation of VSMC proliferation has been considered a key event due to its major implications for the prevention of pathological vascular conditions.^4^

Non‐coding RNAs (ncRNAs) form the dominant product of eukaryotic transcription, comprising over 73% of the human genome.^5^ Circular RNAs (circRNAs) are a special novel type of endogenous ncRNAs, forming covalently closed‐loop structures without 5′ caps and 3′ tails, which make them resistant to degradation by RNA exonuclease and much more stable than linear RNAs.^6^ Recently, studies suggested that circRNAs might be involved in various biological processes via different functional models, including binding microRNAs (miRNAs) as competitive endogenous RNAs (ceRNAs),^7^, ^8^ interacting with RNA binding proteins (RBPs)^9^, ^10^ or coding proteins/peptides.^11^, ^12^, ^13^, ^14^

However, little is known about the roles of circRNAs in vascular biology. The first study showed that circular antisense non‐coding RNA in the INK4 locus (cANRIL) influences the polycomb group (PcG)‐mediated repression of the human INK4a/ARF locus, which is associated with atherosclerosis risk.^15^ CircACTA2 competitively binds miR‐548f‐5p, which suppresses the expression of α‐SMA in VSMCs, and is involved in hypertension.^16^ Our group demonstrated that circ‐Sirt1 controls NF‐κB activation via a sequence‐specific interaction with p65 and enhancement of SIRT1 expression by sponging miR‐132/212 in the inflammatory phenotypic transformation of VSMCs.^17^ Liu et al^18^ found that the expression of circRNA‐ZNF609, a sponge for miR‐615‐5p, has a negative correlation with the velocity of vascular endothelial cell migration and tube formation and is associated with vascular endothelial dysfunction. Besides, other evidence has indicated that the aberrant expression of circRNAs usually accompanies by vascular dysfunction.^19^, ^20^

This study aimed to screen key differentially expressed circRNAs (DEcircRNAs) in proliferative and quiescent human aortic smooth muscle cells (HASMCs). A total of 134 DEcircRNAs were identified, including 66 up‐regulated and 68 down‐regulated circRNAs. Seventy‐seven DEcircRNAs were predicted to be bound with Argonaute (AGO), and five of them were selected to construct the DEcircRNA‐miRNA‐DEmRNA crosstalk network. Gene Ontology (GO) and pathway analyses suggested that hsa_circ_0002579, hsa_circ_0004872, hsa_circ_0006371 and hsa_circ_0040705 mainly participated in the regulation of cell growth pathways. Out of 134 DEcircRNAs, 27 DEcircRNAs with coding potential were screened. Knockdown of hsa_circ_0002579 could inhibit proliferation of HASMCs. Our study provides new clues for exploring the regulation functions of circRNAs in HASMC proliferation and vascular remodelling diseases.

## 2 MATERIALS AND METHODS

### 2.1 Cell culture

Human aortic smooth muscle cells (ScienCell) were cultured in Smooth Muscle Cell Medium (SMCM, ScienCell) with 2% foetal bovine serum (FBS) (ScienCell), 1% 100 × Smooth Muscle Cell Growth Supplement (ScienCell) and 1% 100 × Penicillin/Streptomycin Solution (ScienCell) in a humidified incubator at 37°C containing an atmosphere of 95% air and 5% CO_2_. Quiescent HASMCs were induced by FBS starvation for 24 hours, and proliferative HASMCs were obtained from quiescent HASMCs after treatment with 10 ng/mL PDGF‐BB (R&D Systems Inc) for 24 hours.

### 2.2 RNA labelling and array hybridization

Sample labelling and array hybridization were performed according to the Agilent One‐Color Microarray‐Based Gene Expression Analysis protocol (Agilent Technologies) with minor modifications. Briefly, total RNA was digested with RNase R (Epicentre) to remove linear RNAs and enrich circular RNAs. Then, each sample was amplified and transcribed into fluorescent cRNAs along the entire length of the transcripts without 3′ bias utilizing a random priming method. The labelled cRNAs were purified by the RNeasy Mini Kit (QIAGEN). The concentration and specific activity of the labelled cRNAs (pmol Cy3/μg cRNA) were measured by a NanoDrop ND‐1000 (Thermo Fisher Scientific). Each labelled cRNA (1 μg) was fragmented by adding 5 μL 10 × blocking agent and 1 μL 25 × fragmentation buffer before being heated at 60°C for 30 minutes, followed by the addition of 25 μL 2 × GE Hybridization buffer. Hybridization solution (50 μL) was dispensed into the gasket slide and assembled on the circRNA expression microarray (8x15K, Arraystar) slide. The slides were incubated for 17 hours at 65°C in an Agilent Hybridization Oven. The hybridized arrays were washed, fixed and scanned using the Agilent DNA Microarray Scanner (G2505C). The analysis was conducted by Kangchen Bio‐tech.

Agilent Feature Extraction software (version 11.0.1.1) was used to analyse the acquired array images. Quantile normalization and subsequent data processing were performed using the GeneSpring GX v11.5.1 software package (Agilent Technologies). After quantile normalization of the raw data, circRNAs with at least one out of two samples having flags in Present or Marginal (‘All Targets Value’) were chosen for further data analysis. DEcircRNAs were identified through fold change filtering. Heat map and hierarchical clustering were performed using Agilent GeneSpring GX software (version 11.5.1).

### 2.3 Microarray analysis

An Arraystar Human circRNA Microarray was designed for the global profiling of human circRNAs. Approximately 5816 circRNAs could be detected by the microarrays. Microarray analysis was performed by Kangchen Bio‐tech. The microarray data discussed in this paper have been deposited in NCBI Gene Expression Omnibus and are accessible with the GEO Series accession number http://www.ncbi.nlm.nih.gov/geo/query/acc.cgi?acc=GSE77278 (https://www.ncbi.nlm.nih.gov/geo/query/acc.cgi?acc=GSE77278).

### 2.4 Quantitative real‐time PCR validation

Total RNA was isolated from proliferative and quiescent HASMCs using TRIzol reagent (Thermo Fisher Scientific), and its quantity and quality were examined by a NanoDrop ND‐1000 (Thermo Fisher Scientific) and 1% agarose gel electrophoresis. Then, total RNA was reverse‐transcribed using reverse transcriptase with random primers according to the manufacturer's instructions of the M‐MLV First Strand Kit (TaKaRa). The expression of 13 DEcircRNAs was tested by qRT‐PCR using SYBR Green assays of SuperReal PreMix Plus (TaKaRa), and the primers used for validating up‐ or down‐regulated circRNAs are displayed in Table S1. The qRT‐PCR conditions were as follows: a denaturation step of 3 minutes at 95°C, followed by 40 cycles of 30 seconds at 93°C, 30 seconds at 55°C (adjusted with the Tm of different circRNAs), 20 seconds at 72°C and a final step of 5 minutes at 72°C. All samples in this study were normalized to the internal control β‐actin. The comparative CT (2^−ΔΔCT^) method was used to calculate the fold change of circRNA expression levels, and Student's *t* test was used to test its statistical significance. All primer pairs for the detection of circRNAs were designed against the circRNA‐specific back‐splice sites (Table S1).

### 2.5 Construction of the DEcircRNA‐miRNA‐DEmRNA crosstalk network

Five AGO‐bound DEcircRNAs (hsa_circ_0000069, hsa_circ_0002579, hsa_circ_0004872, hsa_circ_0006371 and hsa_circ_0040705) were selected to construct the interaction network. DEmRNAs were obtained from NCBI Gene Expression Omnibus http://www.ncbi.nlm.nih.gov/geo/query/acc.cgi?acc=GSE77279 (https://www.ncbi.nlm.nih.gov/geo/query/acc.cgi?acc=GSE77279). Shared miRNAs in DEcircRNAs and DEmRNAs were predicted using Arraystar's homemade miRNA target prediction software based on TargetScan^21^ and MiRanda.^22^ The DEcircRNA‐miRNA‐DEmRNA interaction network was illustrated using Cytoscape_v3.7.1.

### 2.6 Gene ontology and pathway analyses

Gene ontology and pathway analyses were performed using the standard enrichment computation method, which was used to determine the potential roles of DEcircRNAs in cellular activities. Based on the online software GeneCodis (http://genecodis.cnb.csic.es/analysis), GO classification and Kyoto Encyclopedia of Genes and Genomes (KEGG) pathway enrichment were conducted for the target genes that might be regulated by these five DEcircRNAs as miRNA sponges in the proliferative and quiescent HASMCs. Gene ontology and pathway analyses are effective methods to uncover the underlying biological function in response to abnormally expressed genes and proteins.^23^ This analysis was used to determine the biological pathways that had significant enrichment of differentially expressed targeted genes.

### 2.7 SiRNA transfection

SiRNA‐hsa_circ_0002579 and siRNA‐control were designed and purchased from GenePharma, China. The sequence of siRNA‐hsa_circ_0002579 was as follows: 5′‐UCCUGUUCAUGGGCUCCAUTT‐3′ and 5′‐AUGGAGCCCAUGAACAGGATT‐3′ and that of siRNA‐control was as follows: 5′‐UUCUCCGAACGUGUCACGUTT‐3′ and 5′‐ACGUGACACGUUCGGAGAATT‐3′. SiRNA‐hsa_circ_0002579 or siRNA‐control was diluted by DEPC water to form a solution of 20 μM for 30 minutes at 4°C. According to the protocol of the HiperFect Transfection Kit (QIAGEN), HiperFect transfection reagent or siRNA was diluted in a serum‐free medium for 5 minutes at room temperature and then HiperFect transfection reagent and siRNA were equal‐ratio mixed for 30 minutes at room temperature to form a polyplex. The polyplex was finally adjusted to a proper volume by the same medium and then added into HASMCs. Following 6 hours of transfection in a humidified incubator at 37°C containing an atmosphere of 95% air, 5% CO_2_, each well containing HASMCs was replaced with the same volume of fresh complete SMCM.

### 2.8 Western blot

Human aortic smooth muscle cells after siRNA‐treated for 48 hours were harvested, and total protein was extracted using RIPA buffer (Solarbio) and 1 mM PMSF (Solarbio). Protein concentrations were determined using the Bradford method (Solarbio). Equal amounts of protein samples were separated by SDS‐PAGE and transferred onto a PVDF membrane (Merck) after electroblotting. The membrane was blocked with 5% non‐fat milk in PBS for 2 hours and then was incubated with the primary antibodies overnight at 4°C: HMGA2 (1:1000, Proteintech), PCNA (1:1,000, Wanleibio), SM22α (1:1,000, CUSABIO) and GAPDH (1:1,000, Wanleibio). After three 5‐minutes washes with TBS‐tween, the membrane was then incubated with goat anti‐rabbit IgG secondary antibody or rat antimouse secondary antibody (dilution at a 1:2000, Wanleibio) at 37°C for 2 hours. The membranes were visualized by ChemiDoc™ MP Imaging System (BIO‐RAD).

### 2.9 Statistical analysis

All data were analysed using SPSS 21.0 software (IBM). The mean ± SD and independent‐samples *t* test were used in the statistical analysis. A value of *P* < .05 was considered significant.

## 3 RESULTS

### 3.1 Analysis of circRNA expression profiling

**Figure 1:**
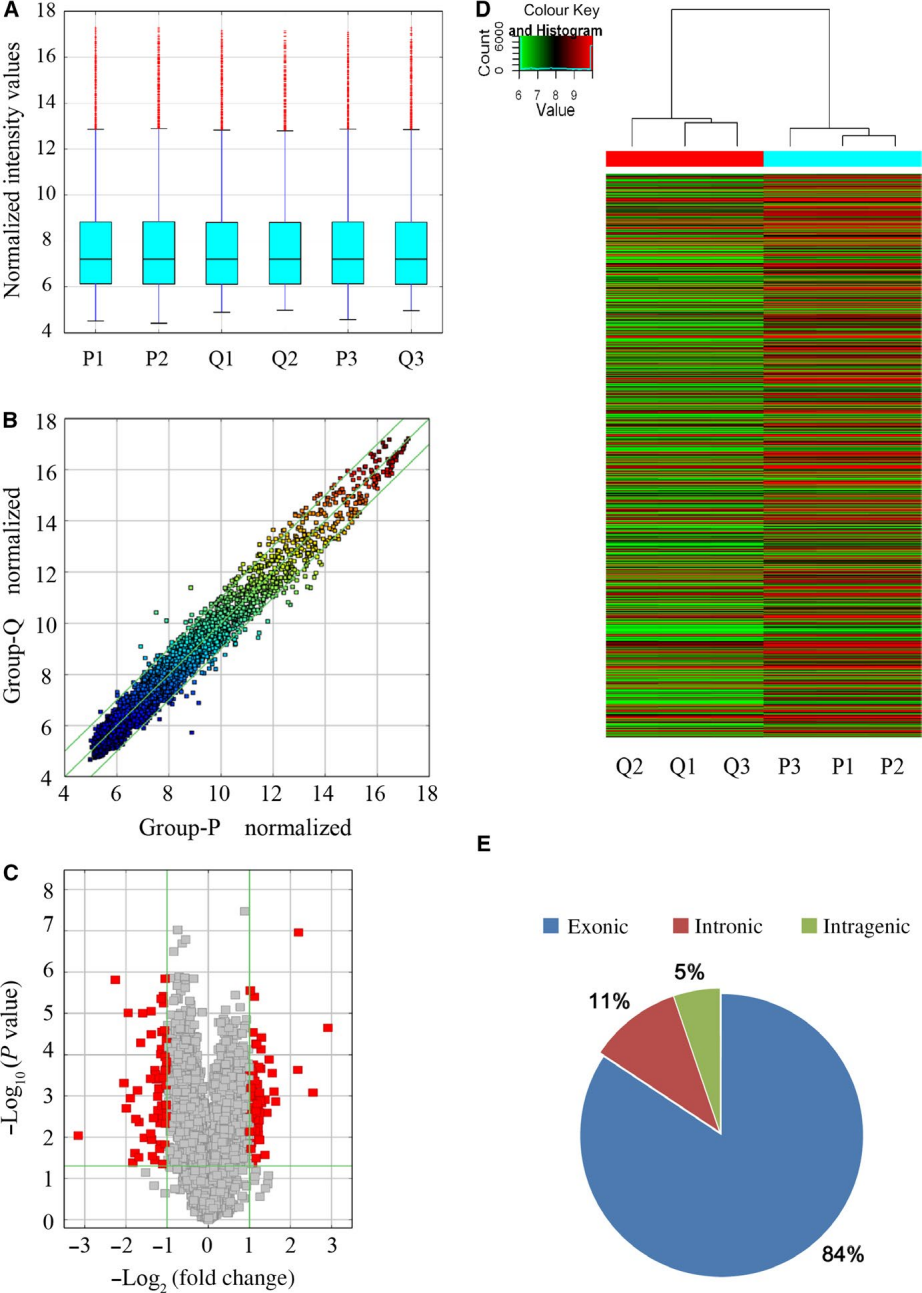
Expression profile of circRNAs in proliferative HASMCs compared to quiescent HASMCs. A, Box plots of circRNAs show the distribution of intensities from all samples. B, Scatter plot showing circRNA expression variation between proliferative HASMCs and quiescent HASMCs. The values of the *X* and *Y* axes in the scatter plots were normalized signal values of the samples (log_2_ scaled). The green lines are fold change lines, with the default fold change value being 2.0. CircRNAs above the top green line and below the bottom green line had more than 2.0‐fold changes between the proliferative HASMCs and quiescent HASMCs. C, Volcano plot showing circRNA differential expression in proliferative HASMCs and quiescent HASMCs. The vertical lines correspond to 2.0‐fold up and down, respectively, and the horizontal line represents a *P*‐value of .05. The red point in the plot represents the differentially expressed RNAs with statistical significance. D, Hierarchical clustering was performed to show differential circRNA expression profiling in proliferative HASMCs and quiescent HASMCs. Cluster analysis arranged samples into groups based on their expression levels, which allowed us to hypothesize the relationships among samples. ‘Red’ denotes high relative expression, and ‘green’ denotes low relative expression. E, The exonic circRNAs account for the majority of the differential circRNAs after being classified

The microarray was applied to detect circRNAs in proliferative and quiescent HASMCs. For the distribution of the datasets, there were no distinct differences among the samples (Figure 1A). The circRNAs that above the top green line and below the bottom green line in the scatter plot (Figure 1B), the red point in the volcano plot (Figure 1C), and the hierarchical clustering (Figure 1D) represent the DEcircRNAs with statistical significance. Among the detectable circRNAs, there were 134 DEcircRNAs in total, including 68 with up‐regulated expression and 66 with down‐regulated expression with a >2‐fold change and a *P*‐value < .05. These 134 DEcircRNAs were classified into three types: 84% were exonic, 11% were intronic and 5% were intragenic (Figure 1E). What is more, we also used circbank^24^ to analyse the conservation of the circRNAs of 134 DEcircRNAs and obtained 41 DEcircRNAs with conservation in humans and mice (Table S2).

### 3.2 Validation by qRT‐PCR

**Figure 2:**
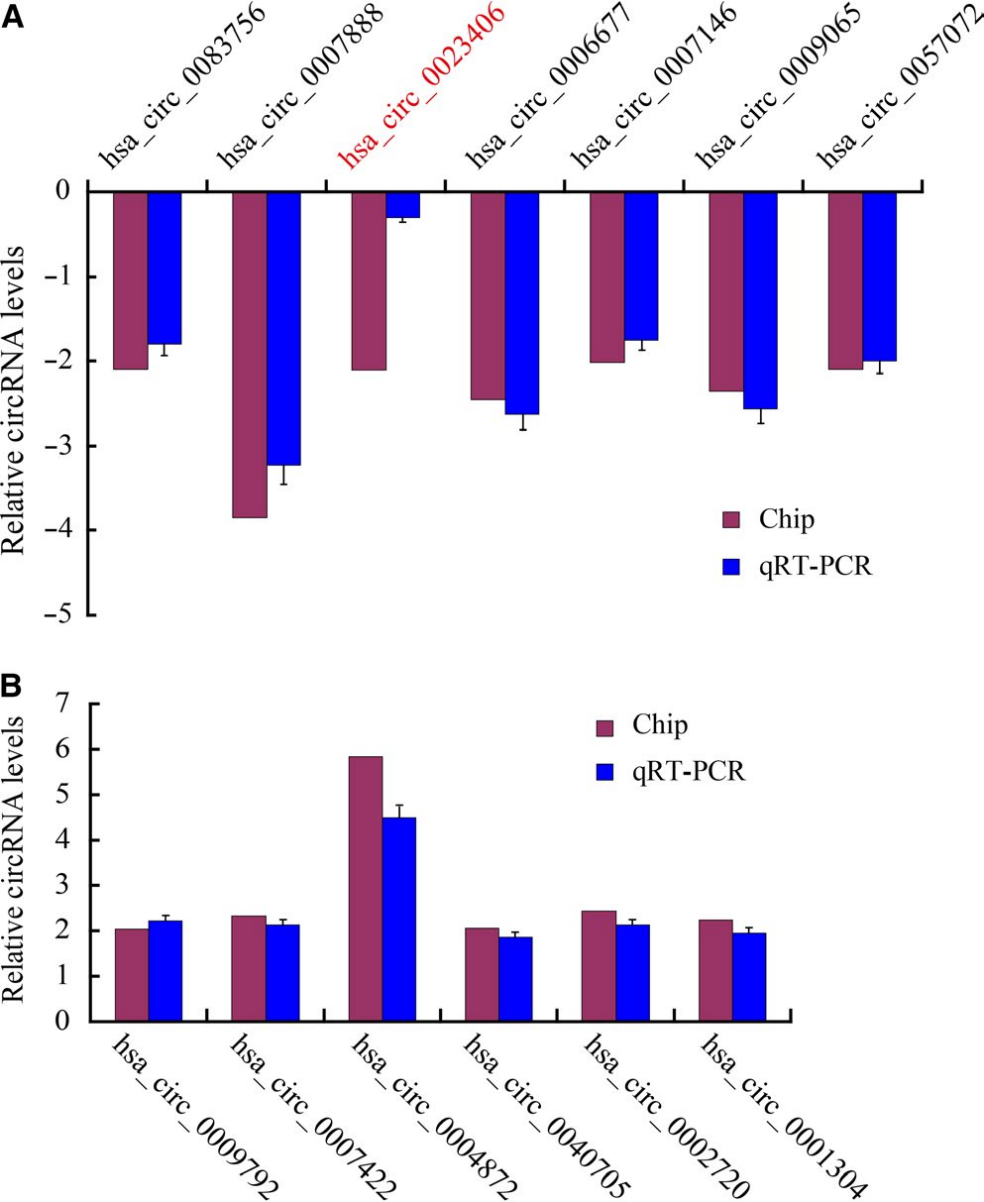
Validation of the DEcircRNAs. The expression levels of 13 DEcircRNAs were validated in the samples of HASMCs by qRT‐PCR and normalized to the internal reference gene β‐actin. A, Apart from hsa_circ_0023406, six verified down‐regulated circRNAs that were consistent with the microarray results. B, Six verified up‐regulated circRNAs that were consistent with the microarray results. All experiments were replicated three times. The presented values are the mean ± SEM. *P < .05

To validate the data accuracy of DEcircRNAs in the microarray, we randomly selected six up‐regulated and seven down‐regulated circRNAs and detected them by qRT‐PCR. As shown in Figure 2A, hsa_circ_0083756, hsa_circ_0007888, hsa_circ_0006677, hsa_circ_0007146, hsa_circ_0009065 and hsa_circ_0057072 were down‐regulated in proliferative HASMCs compared to quiescent HASMCs, except hsa_circ_0023406; and in Figure 2B, hsa_circ_0009792, hsa_circ_0007422, hsa_circ_0004872, hsa_circ_0040705, hsa_circ_0002720 and hsa_circ_0001304 were up‐regulated in proliferative HASMCs compared to quiescent HASMCs.

### 3.3 Construction of the DEcircRNA‐miRNA‐DEmRNA crosstalk network

**Figure 3:**
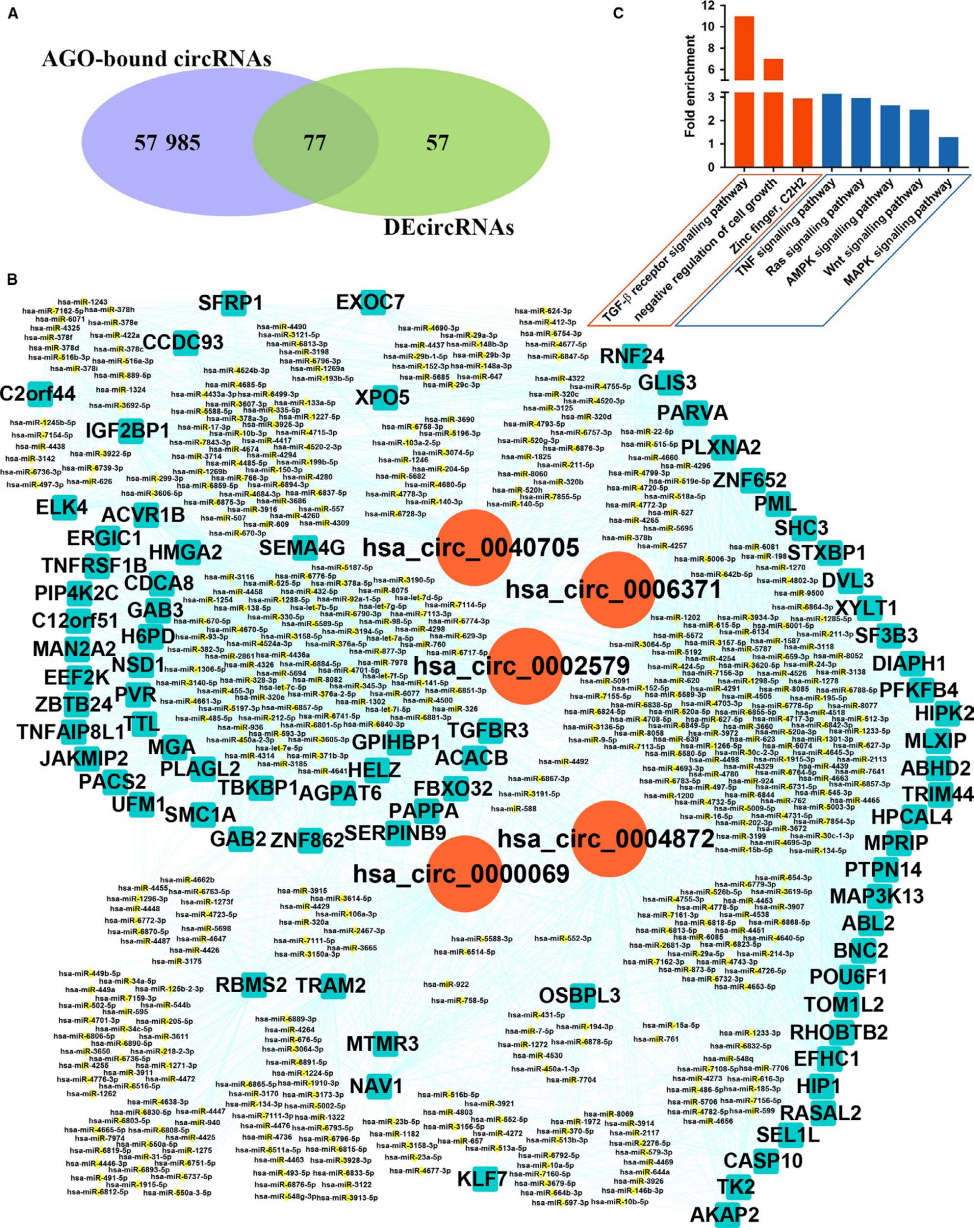
DEcircRNA‐miRNA‐DEmRNA crosstalk network. A, Venn diagram showing that 77 DEcircRNAs were AGO‐bound circRNAs. B, Based on the data of DEcircRNAs and relevant mRNAs in the microarray results mentioned above, 5 AGO‐bound DEcircRNAs (hsa_circ_0000069, hsa_circ_0002579, hsa_circ_0004872, hsa_circ_0006371 and hsa_circ_0040705) were selected to construct the DEcircRNA‐miRNA‐DEmRNA crosstalk network. Red circles: DEcircRNAs. Cyan squares: DEmRNAs. Yellow rhombus: DEmiRNAs. C, Gene Ontology (GO) and pathway analysis of DEmRNAs targeted by five AGO‐bound DEcircRNAs. The three orange ones are GO terms for the DEmRNAs. The five blue ones are pathway terms for the DEmRNAs

As many as 58 062 circRNAs from the circBase database were found to have AGO‐bound regions,^25^ indicating that circRNAs might function as miRNA sponges. Among the 134 DEcircRNAs, 77 DEcircRNAs were predicted to bind with AGO (Figure 3A, Table S3), and five AOG‐bound DEcircRNAs (hsa_circ_0000069, hsa_circ_0002579, hsa_circ_0004872, hsa_circ_0006371 and hsa_circ_0040705) were selected to construct the DEcircRNA‐miRNA‐DEmRNA crosstalk network (Figure 3B). From the interaction network information, we noted that hsa_circ_0002579 shared the miRNA response elements (MREs) of hsa‐miR‐let‐7 with TGFBR3, ACVR1B and GAB2. Hsa_circ_0004872 had the MREs of hsa‐miR‐424‐5p, which were also observed in RASAL2 and ABL2. The DEcircRNA hsa_circ_0006371 shared the MREs of hsa‐miR‐424‐3p, hsa‐miR‐24‐3p and hsa‐miR‐1298‐5p with MAP3K13, shared the MREs of hsa‐miR‐24‐3p with ACVR1B, shared the MREs of hsa‐miR‐424‐5p with ABL2 and shared the MREs of hsa‐miR‐24‐3p and hsa‐miR‐424‐5p with DVL3. Hsa_circ_0040705 shared the MREs of hsa‐miR‐204‐5p with ELK4. Overall, hsa_circ_0000069, hsa_circ_0002579, hsa_circ_0004872, hsa_circ_0006371 and hsa_circ_0040705 were predicted to compete with 2, 35, 16, 28 and 10 DEmRNAs for sponging miRNAs, respectively.

### 3.4 Functional annotation of DEmRNAs targeted by DEcircRNAs

Gene ontology and KEGG pathway analyses were employed to annotate the functions of DEmRNAs that shared the same MREs with five AGO‐bound DEcircRNAs in proliferative and quiescent HASMCs (Figure 3C). The GO analysis results showed that DEmRNAs mainly participated in some significant biological processes and cellular components, such as the TGF‐β receptor signalling pathway, negative regulation of cell growth and zinc finger. Among those significantly enriched GO terms, the TGF‐β receptor signalling pathway (biological process: 0007179) and negative regulation of cell growth (biological process: 0030308) were noteworthy. TGFBR3 shared the same MREs with hsa_circ_0002579, and ACVR1B shared the same MREs with hsa_circ_0002579 and hsa_circ_0006371, which were within those two terms.

According to the KEGG pathway analysis, the Ras signalling pathway (KEGG: cfa04014), TNF signalling pathway (KEGG: cfa04668), AMPK signalling pathway (KEGG: cfa04152), Wnt signalling pathway (KEGG: cfa04310) and MAPK signalling pathway (KEGG: cfa04010) were significantly enriched pathways of interest (Figure 3C). RASAL2, GAB2, SHC3 and ABL2 were enriched in the Ras signalling pathway. RASAL2 and GAB2 were targets of hsa_circ_0004872 and hsa_circ_0002579, respectively, and these two circRNAs regulated the gene ABL2. SHC3 was the target of hsa_circ_0006371. Two genes were enriched in the TNF signalling pathway, including the hsa_circ_0004872‐targeted gene CASP10 and the hsa_circ_0002579‐targeted gene TNFRSF1B; the AMPK signalling pathway including the hsa_circ_0006371‐targeted gene PFKFB4 and the hsa_circ_0002579‐targeted gene EEF2K; the Wnt signalling pathway, including the hsa_circ_0006371‐targeted gene DVL3 and the hsa_circ_0040705‐targeted gene SFRP1; and the MAPK signalling pathway, including the hsa_circ_0040705‐targeted gene ELK4 and the hsa_circ_0006371‐targeted gene MAP3K13.

### 3.5 Coding potential of DEcircRNAs

Through the circRNADb,^26^ we screened 27 DEcircRNAs with internal ribosomal entry sites (IRES) and open reading frames (ORF), suggesting that they might code proteins (Table 1).

**Table 1:** DEcircRNAs with coding potential

circRNA	Chrom	Strand	Gene symbol	Genomic length	Spliced length	Start position	End position	Protein length
hsa_circ_0088688	chr9	+	STXBP1	5195	469	436	2r + 22	174 aa
hsa_circ_0108663	chr18	−	EPB41L3	27 345	1744	224	1r + 30	516 aa
hsa_circ_0138079	chr9	+	SPTAN1	5882	1102	1098	2r + 51	385 aa
hsa_circ_0102717	chr14	+	NRXN3	5765	398	27	1r + 33	134 aa
hsa_circ_0001384	chr3	−	BDH1	23 847	452	44	1r + 29	145 aa
hsa_circ_0000504	chr13	−	TUBGCP3	11 045	751	73	1r + 29	235 aa
hsa_circ_0008462	chr21	−	SYNJ1	7820	665	96	1r + 66	211 aa
hsa_circ_0008512	chr17	−	ULK2	9209	944	889	2r + 65	354 aa
hsa_circ_0008449	chr1	−	GDAP2	12 231	460	95	1r + 54	139 aa
hsa_circ_0139157	chr9	+	NTRK2	88 275	728	117	1r + 120	243 aa
hsa_circ_0092831	chr10	−	UPF2	22 385	959	73	1r + 67	317 aa
hsa_circ_0079509	chr7	−	SNX13	60 957	1624	1563	2r + 51	578 aa
hsa_circ_0108090	chr18	−	OSBPL1A	3267	416	19	1r + 46	147 aa
hsa_circ_0065149	chr3	−	SETD2	5526	854	794	2r + 330	414 aa
hsa_circ_0004619	chr1	−	FAF1	89 334	377	341	2r + 21	144 aa
hsa_circ_0003598	chr15	+	PDIA3	1783	238	223	2r + 61	104 aa
hsa_circ_0125090	chr4	+	ANK2	34 768	1859	12	1r + 15	620 aa
hsa_circ_0006188	chr1	−	GDAP2	6743	383	95	1r + 23	103 aa
hsa_circ_0049629	chr19	−	FARSA	3554	301	150	2r + 21	157 aa
hsa_circ_0002092	chr2	−	NCKAP1	927	448	14	1r + 27	153 aa
hsa_circ_0057262	chr2	−	NCKAP1	18 580	706	14	1r + 27	239 aa
hsa_circ_0065140	chr3	−	SETD2	24 681	1241	312	1r + 330	419 aa
hsa_circ_0121581	chr3	−	GSK3B	23 374	325	152	2r + 83	193 aa
hsa_circ_0119391	chr2	+	LRRFIP1	20 974	904	127	1r + 110	295 aa
hsa_circ_0108320	chr18	+	DTNA	15 767	397	2	1r + 9	134 aa
hsa_circ_0069853	chr4	+	POLR2B	9056	716	3	1r + 69	260 aa
hsa_circ_0110127	chr1	+	AGL	36 809	2870	4	1r + 46	970 aa

Abbreviation: DEcircRNAs, differentially expressed circRNAs.

### 3.6 Comparison of expression levels between DEcircRNAs and corresponding linear mRNAs

**Figure 4:**
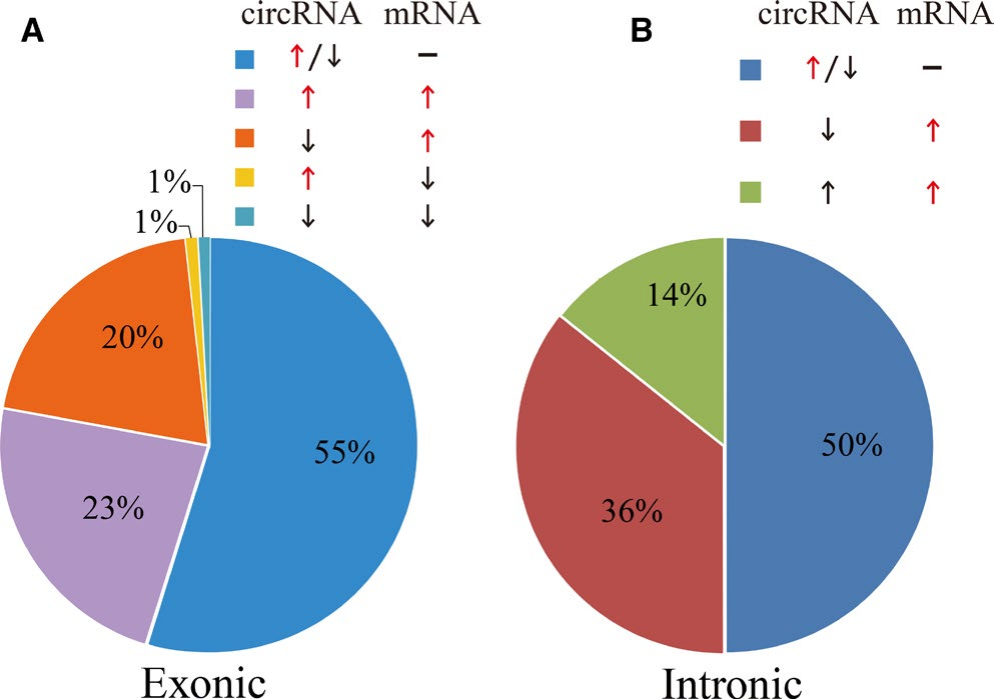
Comparison of expression levels of exonic and intronic DEcircRNAs to corresponding linear RNAs in proliferative HASMCs and quiescent HASMCs. Arrows represent the change of expression level. Upward arrows stand for the up‐expression; downward arrows stand for down‐expression, and the transverse lines stand for the invariable expression. A, Five categories, including co‐expression (up‐regulation or down‐regulation), inverse‐expression (circRNAs downward and mRNA upward or circRNAs upward and mRNA downward) and irrelevant expression, were based on the exonic DEcircRNAs and the corresponding mRNAs. B, There are only three categories in the intronic circRNAs. The relevant and irrelevant expressions are fifty‐fifty

The main two categories of DEcircRNAs were exonic and intronic. No matter in which portion, there were less than half of the corresponding mRNAs whose expression was changing along with the differential expression of the circRNAs. In the part of exonic circRNAs, there were 62 circRNAs irrelated with their host genes, 26 co‐expression (co‐up or co‐down) and 23 inverse‐expression (circRNAs downward while corresponding mRNAs upward or circRNAs upward while corresponding mRNAs downward) (Figure 4). Only a small moiety of the DEcircRNAs derived from introns and half of them have nothing to do with the corresponding mRNAs (Figure 4).

### 3.7 Knockdown of hsa_circ_0002579 inhibited the proliferation of HASMCs

**Figure 5:**
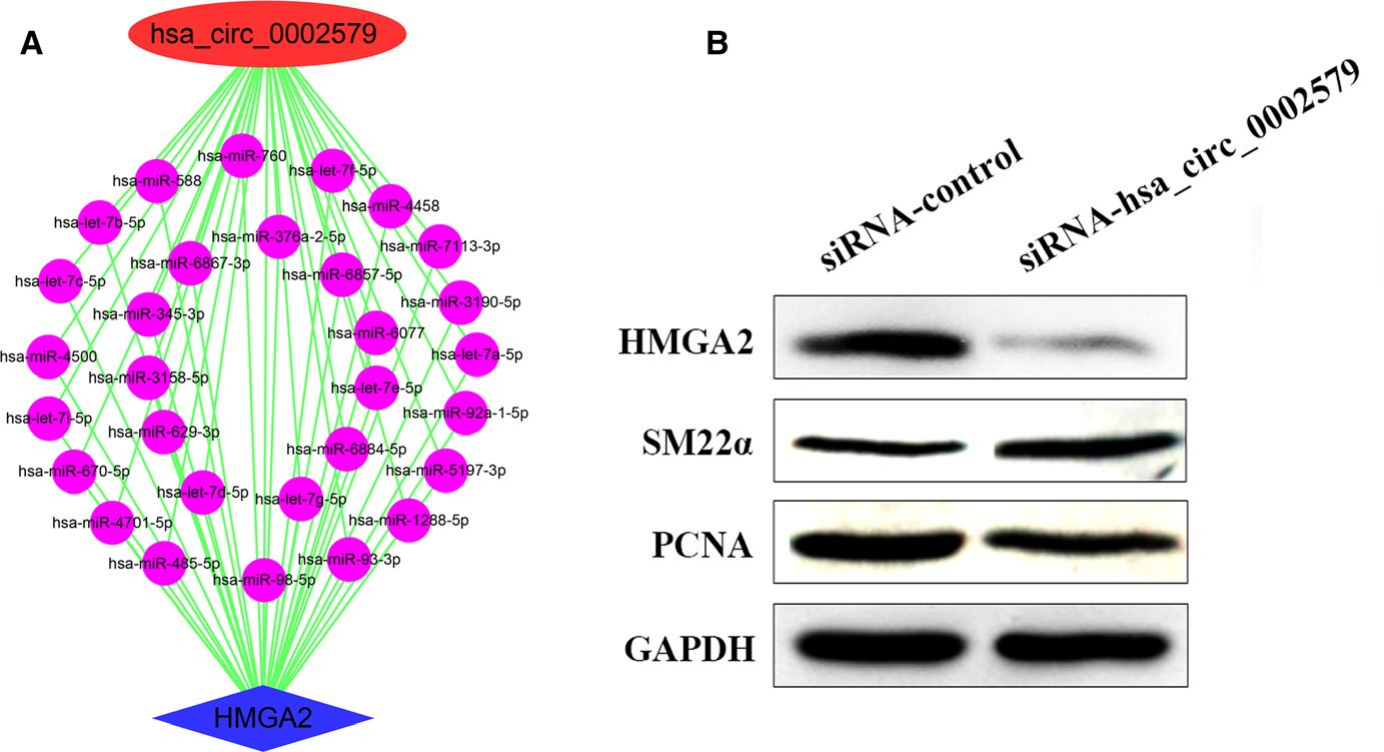
Knockdown of hsa_circ_0002579 inhibited the proliferation of HASMCs. A, Thirty shared miRNAs between hsa_circ_0002579 and HMGA2 3′‐UTR. B, HASMCs were treated with siRNA‐hsa_circ_0002579/siRNA‐Control for 48 h. Cell lysates were extracted and subjected to Western blotting using specific antibodies against HMGA2, PCNA and SM22α. GAPDH was used as equal loading control. The illustrated results were representative of three independent experiments

From the data obtained by microarray, we found that hsa_circ_0002579 was up‐regulated in the proliferative HASMCs. Gene ontology and pathway analyses showed that 35 DEmRNAs co‐expressed with hsa_circ_0002579 (Figure 3B) were enriched in the TGF‐β receptor signalling pathway, Ras signalling pathway and AMPK signalling pathway (Figure 3C). Interestingly, high mobility group AT‐hook 2 (HMGA2) is one of the DEmRNAs that co‐expressed with hsa_circ_0002579, 30 shared miRNAs were found between hsa_circ_0002579 and HMGA2 3′‐UTR (Figure 5A), indicating that hsa_circ_0002579 might target HMGA2 as ceRNA. SiRNA for hsa_circ_0002579 was designed, which could decrease 65% hsa_circ_0002579 levels (data not shown). Compared to control, knockdown of hsa_circ_0002579 reduced the expression level of HMGA2 and PCNA and increased the expression level of SM22a (Figure 5B).

Taken together, these data showed that knockdown of hsa_circ_0002579 could inhibit the proliferation of HASMCs. There was a hint that hsa_circ_0002579 could be a promising ceRNA, and HMGA2 was one of its downstream targets.

## 4 DISCUSSION

CircRNAs, mainly produced by precursor mRNA backsplicing of exons, have recently been predicted as a novel class of gene expression potential regulatory factors. CircRNAs often show cell‐type‐specific, tissue‐specific and spatiotemporal‐specific patterns, including the cardiovascular system,^27^ and multitudinous studies suggested that circRNAs are associated with CVD.^15^, ^16^, ^18^, ^19^, ^20^ However, direct evidence about circRNAs in VSMCs in terms of proliferation regulation is still absent. Since the ceRNA hypothesis was proposed,^28^ emerging evidence has indicated that circRNAs could act as miRNA sponges to regulate the stability or translation of mRNAs. In this study, we aimed to explore the roles of circRNAs acting as ceRNAs to mediate the proliferation of VSMCs. Here, we performed microarray analysis to identify 134 DEcircRNAs in proliferative HASMCs compared to quiescent HASMCs, and we predicted 77 AGO‐bound DEcircRNAs that were promising ceRNA molecules. And hsa_circ_0000069, hsa_circ_0002579, hsa_circ_0004872, hsa_circ_0006371 and hsa_circ_0040705 were selected to construct the DEcircRNA‐miRNA‐DEmRNA interaction network to predict their functions.

According to recent significant research, many miRNAs, including miR‐24‐3p,^29^ miR‐424‐5p,^30^ miR‐1298‐5p,^31^ miR‐204‐5p^32^ and let‐7a‐5p,^33^ are involved in VSMC proliferation and down‐regulated in proliferative VSMCs. In the HASMCs, the MREs of the miRNAs mentioned above were predicted in DEcircRNAs. We expected that the expression of DEcircRNA‐sponged miRNAs was suppressed when these circRNAs were up‐regulated in proliferative HASMCs.

Here, we found that hsa_circ_0002579 shared the same MREs as hsa‐let‐7a‐5p with GAB2, TGFBR3 and ACVR1B. GAB2 was annotated in the Ras signalling pathway, an important pathway involved in cell proliferation. Thus, hsa_circ_0002579 might be a promising mediator of HASMC proliferation via involvement in the Ras signalling pathway. The well‐known TGF‐β signalling pathway is involved in many cellular processes of VSMCs, including cell growth. TGFBR3 is the most abundant TGF‐β type II receptor, and the impact of TGFBR3 on TGF‐β signalling depends on the circumstances.^34^ Our results suggested that hsa_circ_0002579 might be involved in the TGF‐β signalling pathway to regulate HASMC proliferation through sponging hsa‐let‐7a‐5p. ACVR1B, one type I receptor in the TGF‐β signalling pathway, was expected to share MREs of hsa‐let‐7a‐5p with hsa_circ_0002579 and share MREs of hsa‐miR‐24‐3p with hsa_circ_0006371. MiR‐24‐3p was shown to participate in the TGF‐β signalling pathway and Wnt signalling pathway to regulate the growth of VSMCs.^35^, ^36^ Hence, hsa_circ_0002579 and hsa_circ_0006371 may synergistically regulate HASMC proliferation through the TGF‐β signalling pathway. In terms of the Wnt signalling pathway, we showed that DVL3 shares strong similarity with DSH, functioning as a principal component of the Wnt signalling pathway and governing several cellular processes, including cell proliferation and differentiation.^37^ DVL3 shared the MREs of hsa‐miR‐24‐3p and hsa‐miR‐424‐5p with hsa_circ_0006371 in HASMCs. One signalling modulator, Rspo3, of the Wnt signalling pathway was identified as a potential regulator of coronary stem proliferation.^37^ Additionally, the Wnt signalling pathway was shown to be involved in the proliferation and migration of pulmonary arteriolar smooth muscle cells.^38^ Consequently, we conjecture that hsa_circ_0006371 would be a positive conductor of Wnt signals isolating hsa‐miR‐24‐3p and hsa‐miR‐424‐5p to regulate HASMC proliferation.

In vitro, the overexpression of miR‐424‐5p inhibited VSMC proliferation,^30^ which implies that miR‐424‐5p might be induced as a signal to counteract the proliferation. The MREs of hsa‐miR‐424‐5p were predicted in hsa_circ_0006371 and hsa_circ_0004872, which were also predicted in the DEmRNA ABL2. ABL2 was annotated in the Ras signalling pathway. Some researchers have identified ABL2 as the target of many regulators to facilitate the proliferation process.^39^ Here, we proposed that hsa_circ_0006371 and hsa_circ_0004872 could sponge hsa‐miR‐424‐5p to up‐regulate ABL2 to be involved in the proliferation of HASMCs via the Ras signalling pathway. Moreover, hsa_circ_0006371 shared the same MREs of hsa‐miR‐24‐3p, hsa‐miR‐424‐5p and hsa‐miR‐1298‐5p with MAP3K13 (namely LZK). The JNK signalling pathway was downstream of MAP3K13. A specific role of miR‐1298 in regulating VSMC proliferation was demonstrated.^31^ The MAPK/JNK pathway also might regulate VSMC proliferation.^40^ In addition, another DEmRNA annotated in the MAPK pathway was ELK4, an ERK‐regulated cofactor of the SRF transcription factor, which has been shown to act as a target of the ERK, JNK and p38 MAPK families.^41^, ^42^, ^43^, ^44^ Based on the network, we predicted that ELK4 shared the MREs of hsa‐miR‐204‐5p with hsa_circ_0040705. This implied that hsa_circ_0006371 and hsa_circ_0040705 might synergize the proliferation of HASMCs by the MAPK/JNK signalling pathway.

However, the target miRNAs may not necessarily be affected by low levels of circRNAs.^45^ Besides, in experimental verification, it is difficult to simulate the expression of miRNAs and ceRNAs in the physiological state through exogenous miRNAs and ceRNAs.^46^ Furthermore, previous findings have shown that some circRNAs were unable to function as miRNA sponges,^25^ which suggested that circRNAs may exert their effects through other mechanisms, such as forming complexes with proteins^9^, ^10^ directly or translating into proteins/peptides.^11^, ^12^, ^13^, ^14^ Interestingly, for the translation of circRNAs, out of 134 DEcircRNAs, we screened 27 circRNAs with IRES and ORF, indicating the coding potential of these circRNAs (Table 1).

In one previous study, the results showed that circRNAs were typically generated at the cost of canonical mRNA isoforms and even more abundant than the linear counterparts.^47^ Identifying hundreds of head‐to‐tail junction reads in published data sets of chromatin‐bound (nascent) RNA from fly heads,^48^ circRNAs were considered as being generated co‐transcriptionally and could compete with linear splicing mutually.^49^ However, some studies beg to differ. The expression of some circRNAs is found to be independent of related linear isoforms.^50^, ^51^ The specifically overexpressed circRNAs in the multiple system atrophy brain have been determined, and the expression levels of linear transcripts are not significantly altered and thus do not follow the pattern of their circular counterparts.^52^ With the alteration of circRNAs, only a small percentage of the corresponding mRNAs also make a change, which is consistent with that only a few circRNAs show co‐regulation with their host genes, and circRNAs exhibited changes independent of the cognate mRNA.^53^ In our study, we also failed to find the clear‐cut relationship between circRNAs and the cognate linear transcripts. And we expected that the production of most circRNAs is regulated, which might be indispensable in the mediation of gene expression.^54^ Moreover, circRNAs generated from one locus could be several and different. Thus, the relationship of homology circRNAs, as well as respective functions, required further exploration.

By DEcircRNAs‐DEmRNAs co‐expression subnetwork analysis, we found has_circ_0002579 was co‐expression with 35 DEmRNAs in the proliferative HASMCs (Figure 3B), and 30 miRNAs shared by hsa_circ_0002579 and HMGA2 3′‐UTR (Figure 5A). Studies have shown that HMGA2 is overexpressed in cancer cells^55^, ^56^ and promotes angiogenesis.^57^ And miR‐4500,^58^ miR‐98‐5p^59^ and miR‐485‐5p^60^ have been reported to directly target the 3′‐UTR of HMGA2 in other cell‐type studies. These cases profile the bright prospects of hsa_circ_0002579, which acts as a ceRNA molecule interacting with miRNAs to regulate the proliferation of HASMCs by targeting HMGA2. We found that hsa_circ_0002579 was up‐regulated in the proliferative HASMCs (Figure 1), and knockdown of hsa_circ_0002579 could decrease the expression level of HMGA2 and PCNA (Figure 5B). We speculated that hsa_circ_0002579 might cross‐interact with miRNAs to increase the expression of HMGA2, ultimately positively regulating the proliferation of VSMCs. However, further experimental work remains to be done to validate those forecasting results.

In our study, we investigated the DEcircRNAs that were found in the proliferative and quiescent conditions of HASMCs to help elucidate their function. Next, the DEcircRNA‐miRNA‐DEmRNA crosstalk network was constructed for further investigations. Then, we analysed the coding potential of DEcircRNA and the effect of DEcircRNA on the corresponding linear RNA. Finally, hsa_circ_0002579, one of the DEcircRNAs, might target HMGA2 to promote the proliferation of HASMCs by sponging miRNAs, which may be a new therapeutic target for VSMC proliferative vascular diseases. These findings provide novel insights for understanding the functions of circRNAs in HASMC proliferation and vascular remodelling diseases.

## CONFLICT OF INTEREST

The authors declare that the research was conducted in the absence of any commercial or financial relationships that could be construed as a potential conflict of interest.

## AUTHOR CONTRIBUTIONS

SS conceived and designed this study. WC, JL, BL, SC, YL, YL, KL and SS performed the bioinformatics analysis. WC, JL, BL, HL and JZ performed the experiments. WC, JL, SC and SS wrote the manuscript. All authors read and approved the final manuscript.

## ETHICAL APPROVAL

The human aortic smooth muscle cells (HASMCs) we used in this study were commercial cells purchased from ScienCell.

## Supporting information

Table S1

Table S2

Table S3

## Data Availability

Raw sequence reads have been deposited in the GEO database (http://www.ncbi.nlm.nih.gov/geo/query/acc.cgi?acc=GSE77278 and http://www.ncbi.nlm.nih.gov/geo/query/acc.cgi?acc=GSE77279).
