# Supplementary material for: Screening and functional prediction of differentially expressed circRNAs in proliferative human aortic smooth muscle cells
Source: J Cell Mol Med. 2020 Mar 10;24(8):4762–72. doi: 10.1111/jcmm.15150 (PMC7176856; doi:10.1111/jcmm.15150)
Supplement: Supplementary file 1 — Table S1 [file JCMM-24-4762-s001.pdf]

Supplementary Table S1. The primers used in qRT-PCR experiments

DEcircRNAs	Primers sequence (5′ to 3′)
hsa_circ_0083756	Forward：GAGACGGAGGTGCTGGCACATGA
	Reverse：TGGCCTGCTCCTCCACTCTCAAG
hsa_circ_0007888	Forward：CTTTGCGGATTCTCAGTAAATACCACC
	Reverse：CACAGGAGAAAGTACCAGCTGAGCGTC
hsa_circ_0006677	Forward：CGTTAGGGCAGTTTACAAGGTCAGTTTTAGG
	Reverse：ACTTCTTTGGTTGTGTGGCATTGTTCCTGT
hsa_circ_0009065	Forward：GGTTTCCCCAAAGTCAGTATTCTCCTCA
	Reverse：GGTTCATCTCTCTCAAGGTCCATTGTGTTC
hsa_circ_0007146	Forward：CTTGTAAGTGTGAGCACCATTGTCC
	Reverse：ACACACACCACGGTGGACACTT
hsa_circ_0057072	Forward：AGGTCAAGGCTATGGTGCTCAAC
	Reverse：TGGTGTTACATTTGTACTCTGCCAGTTTCT
hsa_circ_0023406	Forward：ATGGTCCTCATCCTCATCGCAAC
	Reverse：TGCATAGCTTCATGGCCACG
hsa_circ_0009792	Forward：GCTCCGGACTATGACCACTTGAC
	Reverse：CAGCCAATGAGGCCCGAGT
hsa_circ_0007422	Forward：CATCCGTCGCTTCCTGAACCTT
	Reverse：ATTCAAGAGAGCCGTCCAACTGC
hsa_circ_0004872	Forward：CCGTGTTGCAGATCCAGACCATG
	Reverse：CAGTAGGTCTGGTGCTCAAAGGGG
hsa_circ_0040705	Forward：CCACTTTGCAGCCATTTTTCACG
	Reverse：GCAACGACACTGGTTTATGOATTAGGG
hsa_circ_0002720	Forward：CCGAGCCACGCATTTACATCAGT
	Reverse：TTGGCCTTGAAAGTCCCAGATGG
hsa_circ_0001304	Forward：CATCCGCCTCATAAAAGACAAACAGAC
	Reverse：CCTGTTTTCCTCTTCATCAGCCTCAC
has−β−actin	Forward：GACTTAGTTGCGTTACACCCTTTCTTG
	Reverse：ACTGCTGTCACCTTCACCGTTCC

qRT-PCR: quantitative real-time polymerase chain reaction

DEcircRNAs: differentially expressed circRNAs
