# Supplementary material for: Screening and functional prediction of differentially expressed circRNAs in proliferative human aortic smooth muscle cells
Source: J Cell Mol Med. 2020 Mar 10;24(8):4762–72. doi: 10.1111/jcmm.15150 (PMC7176856; doi:10.1111/jcmm.15150)
Supplement: Supplementary file 2 — Table S2 [file JCMM-24-4762-s002.pdf]

Supplementary Table S2. DEcircRNAs with circRNA conservation

DEcircRNAs (up)	Conserved mouse circRNAs	DEcircRNAs (down)	Conserved mouse circRNAs
hsa_circ_0000520	mmu_circ_0000536	hsa_circ_0011120	mmu_circ_0011284
hsa_circ_0009581	mmu_circ_0011447	hsa_circ_0007895	mmu_circ_0001277
hsa_circ_0002720	chr4_136693734_136696154_-	hsa_circ_0007364	chr4_129838977_129842762_+
hsa_circ_0002454	mmu_circ_0010983	hsa_circ_0020097	mmu_circ_0000967
hsa_circ_0006371	chr19_41552448_41555701_+	hsa_circ_0000284	mmu_circ_0001052
hsa_circ_0003110	mmu_circ_0010255	hsa_circ_0000369	chr9_32461250_32476773_-
hsa_circ_0032704	mmu_circ_0004297	hsa_circ_0038111	chr16_14419699_14423260_+
hsa_circ_0034326	mmu_circ_0014342	hsa_circ_0046123	mmu_circ_0002826
hsa_circ_0003838	chr2_120760196_120777599_-	hsa_circ_0060733	chr2_166921969_166922844_+
hsa_circ_0036399	chr9_55482272_55488929_-	hsa_circ_0002657	mmu_circ_0005921
hsa_circ_0000665	chr17_25264765_25269479_-	hsa_circ_0001246	mmu_circ_0005924
hsa_circ_0002598	mmu_circ_0003485	hsa_circ_0067772	mmu_circ_0010754
hsa_circ_0046599	mmu_circ_0002856	hsa_circ_0001402	chr5_64311058_64324609_+
hsa_circ_0046600	chr11_121639751_121651673_-	hsa_circ_0004136	chr1_21535060_21593614_-
hsa_circ_0049392	chr9_21742186_21742796_	hsa_circ_0004365	mmu_circ_0012389
hsa_circ_0003218	mmu_circ_0008831	hsa_circ_0001741	mmu_circ_0013256
hsa_circ_0059702	mmu_circ_0009441	hsa_circ_0083756	chr14_66304004_66304235_+
hsa_circ_0004968	chr16_58424560_58424788_+	hsa_circ_0087641	chr13_64209106_64247318_-
hsa_circ_0081789	mmu_circ_0012433	hsa_circ_0008240	mmu_circ_0009881
hsa_circ_0002702	chr4_43414627_43416718_+		
hsa_circ_0002191	mmu_circ_0004730		
hsa_circ_0089974	chrX_161871468_161875392_		

DEcircRNAs: differentially expressed circRNAs
