# Supplementary material for: Screening and functional prediction of differentially expressed circRNAs in proliferative human aortic smooth muscle cells
Source: J Cell Mol Med. 2020 Mar 10;24(8):4762–72. doi: 10.1111/jcmm.15150 (PMC7176856; doi:10.1111/jcmm.15150)
Supplement: Supplementary file 3 — Table S3 [file JCMM-24-4762-s003.pdf]

Supplementary Table S3. 77 AGO-bounded circRNAs

circRNA ID	Chromosome	Start	Stop	Strand
hsa_circ_0004368	chr6	139264649	139265759	-
hsa_circ_0009065	chr16	14738130	14738466	+
hsa_circ_0000520	chr14	20811436	20811559	-
hsa_circ_0005785	chr12	110819556	110834257	-
hsa_circ_0000069	chr1	47745912	47748131	-
hsa_circ_0029605	chr13	20235837	20244503	+
hsa_circ_0001741	chr7	128655032	128658211	-
hsa_circ_0046188	chr17	79555969	79575848	-
hsa_circ_0038111	chr16	16146580	16150152	+
hsa_circ_0000519	chr14	20811436	20811534	-
hsa_circ_0087493	chr9	95018961	95048121	-
hsa_circ_0007364	chr1	32381495	32385259	-
hsa_circ_0038350	chr16	19627435	19702793	+
hsa_circ_0007895	chr1	28362054	28384605	-
hsa_circ_0002579	chr19	11223953	11224438	+
hsa_circ_0011120	chr1	28362054	28369161	-
hsa_circ_0004099	chr11	9225206	9229179	-
hsa_circ_0004565	chr3	149563797	149619949	+
hsa_circ_0002454	chr1	65830317	65831879	+
hsa_circ_0007422	chr2	200173482	200298237	-
hsa_circ_0007018	chr16	69189773	69201088	+
hsa_circ_0068631	chr3	195802029	195803993	-
hsa_circ_0088807	chr9	131271154	131271376	+
hsa_circ_0004365	chr7	80418621	80440017	-
hsa_circ_0032389	chr14	71428942	71462642	+
hsa_circ_0083756	chr8	27151596	27151827	-
hsa_circ_0001246	chr22	46096161	46136418	+
hsa_circ_0002714	chr7	80418621	80435074	-
hsa_circ_0040705	chr16	84792321	84801964	+
hsa_circ_0003692	chr3	171969049	172028671	+
hsa_circ_0006903	chr12	133310970	133312098	-
hsa_circ_0004968	chr3	98600383	98600611	-
hsa_circ_0016404	chr1	212977661	212977993	+
hsa_circ_0059175	chr20	1115763	1115949	+
hsa_circ_0007367	chr9	33948371	33956144	-
hsa_circ_0057072	chr2	172782046	172823470	+
hsa_circ_0064557	chr3	18456602	18462483	-
hsa_circ_0004872	chr22	22153300	22162135	-
hsa_circ_0004870	chr20	34302106	34313077	-
hsa_circ_0020929	chr11	3988781	4080626	+
hsa_circ_0008012	chr4	103446668	103459113	+
hsa_circ_0001147	chr20	34304661	34313077	-
hsa_circ_0092288	chr3	49070226	49070546	-
hsa_circ_0060733	chr20	47682728	47683817	+

hsa_circ_0002940	chr12	69983264	69987393	+
hsa_circ_0059702	chr20	30142548	30149539	+
hsa_circ_0008274	chr13	96485180	96489456	-
hsa_circ_0005483	chr16	929569	961079	-
hsa_circ_0002598	chr17	58700881	58711338	+
hsa_circ_0002657	chr22	46085591	46125470	+
hsa_circ_0000077	chr1	62171487	62175109	-
hsa_circ_0009581	chr1	8555122	8601377	-
hsa_circ_0051732	chr19	48660270	48660397	-
hsa_circ_0079375	chr7	6618131	6624891	+
hsa_circ_0021569	chr11	32948702	32949669	+
hsa_circ_0086740	chr9	34011624	34017187	-
hsa_circ_0075736	chr6	17669523	17669777	-
hsa_circ_0009131	chr3	183454505	183480067	+
hsa_circ_0003945	chr9	33953282	33956144	-
hsa_circ_0006371	chr10	98708764	98711953	+
hsa_circ_0000395	chr12	46622935	46624417	-
hsa_circ_0036399	chr15	76566752	76580286	-
hsa_circ_0004200	chr15	96826060	96834012	-
hsa_circ_0003218	chr2	203329531	203332412	+
hsa_circ_0084429	chr8	48817428	48842572	-
hsa_circ_0002224	chr10	51374369	51387763	+
hsa_circ_0067772	chr3	155547476	155560408	-
hsa_circ_0030741	chr13	100206557	100207901	+
hsa_circ_0000665	chr16	1364020	1370518	+
hsa_circ_0046600	chr17	80962990	80992975	-
hsa_circ_0068610	chr3	195785154	195787118	-
hsa_circ_0087890	chr9	111795586	111812972	-
hsa_circ_0072255	chr5	37326002	37328522	-
hsa_circ_0046599	chr17	80962990	80972395	-
hsa_circ_0001535	chr5	137320945	137324004	-
hsa_circ_0000284	chr11	33307958	33309057	+
hsa_circ_0002702	chr9	35546426	35548532	+

AGO: argonaute.
